# Cholinergic basal forebrain degeneration is associated with central fatigue in Parkinson’s disease

**DOI:** 10.3389/fneur.2025.1682573

**Published:** 2025-11-04

**Authors:** Li Li, Xiaodan Zhang, Mateng Wang, Yuemin Wu, Zhaoying Chen, Yafen Jiang, Timonthy Rainer, Qiongfeng Guan, Weinv Fan

**Affiliations:** ^1^Department of Neurology, Ningbo No.2 Hospital, Ningbo, China; ^2^Department of Emergency Medicine, The University of Hong Kong, Hong Kong, Hong Kong SAR, China; ^3^Department of General Surgery, Yinzhou No.2 Hospital, Ningbo, China

**Keywords:** Parkinson disease, central fatigue, cholinergic system, basal forebrain, grey matter density, grey matter volume, voxel-based morphometry

## Abstract

**Background:**

Central fatigue is a prevalent and debilitating non-motor symptom in Parkinson’s disease (PD), yet its neurobiological basis remains poorly understood. While cholinergic basal forebrain (CBFB) degeneration has been implicated in various PD symptoms, its specific relationship with central fatigue has not been systematically investigated using neuroimaging.

**Methods:**

In this cross-sectional study, 49 patients with PD underwent 3.0 T brain structural magnetic resonance imaging (MRI) and completed the Fatigue Severity Scale (FSS). Gray matter density (GMD) and gray matter volume (GMV) of CBFB subregions [cholinergic nucleus 4 (Ch4) and cholinergic nucleus 123 (Ch123)] were quantified using voxel-based morphometry with validated probabilistic atlases. Multiple linear regression models examined associations between FSS scores and regional GMD/GMV, adjusting for age, sex, and total intracranial volume (TIV).

**Results:**

Fatigue severity showed significant negative correlations with bilateral Ch4 integrity across both morphometric measures. Specifically, higher FSS scores were associated with reduced GMD in total, left, and right Ch4, and with smaller GMV in total and right Ch4. A weaker but significant negative correlation was also observed between fatigue and left Ch123 GMD, whereas no association was found with Ch123 GMV. Crucially, parallel analyses of GMD and GMV yielded convergent results, reinforcing the robustness of the Ch4-fatigue relationship.

**Conclusion:**

This study provides initial neuroimaging evidence linking cholinergic basal forebrain structural integrity with central fatigue in Parkinson’s disease. The consistent associations observed between fatigue severity and bilateral Ch4 morphology, supported by parallel GMD and GMV analyses, suggest a potential role for CBFB degeneration in PD-related fatigue. While further validation is needed, these findings contribute to the growing evidence supporting cholinergic involvement in non-motor symptoms and highlight Ch4 as a promising region for future investigations into fatigue mechanisms.

## 1 Introduction

Fatigue is one of the most common nonmotor manifestations of Parkinson’s disease (PD) (1, 2) and affects up to 50% of the patients during the disease course (3), limiting their ability to maintain hobbies and participate in social activities. It may also predate the onset of motor symptoms in PD (4) and tends to persist over time (5), negatively impacting on patients’ quality of life (6) and daily activities (7). Epidemiological studies have generally not found significant associations between disease duration, stage, or motor symptoms and fatigue (3). Currently insufficient evidence exists to support the treatment of fatigue in PD with any drug or nondrug treatment (8). Therefore, to further investigate the mechanisms of fatigue in PD could have great significance.

Fatigue in PD is mainly central fatigue (9). Regarding CNS causes of fatigue, Chaudhuri and Behan proposed a neuroanatomic model (9, 10) in which disruption of cortical–subcortical loops important for internal drive causes fatigue by altering one’s ability to maintain consistent effort. According to their fatigue model, fatigue relies on incomplete or disconnection of cortical structures and the basal ganglia to maintain performance. Thus, examining the integrity of the corresponding brain networks or brain structure, may be an important direction for further research in PD patients with central fatigue. Recent structural and functional neuroimaging studies on PD patients with fatigue have confirmed that PD with fatigue may be associated with gray matter volume (GMV) reduction in frontal, parietal, insula and other brain regions, global cortical atrophy, white matter injury, metabolic abnormalities in multiple brain regions, and changes in functional connectivity of sensorimotor networks (11–15).

In the context of PD pathology, there is little doubt that multiple pathologies can affect the pallido-thalamic input to the frontal cortex. Biochemical changes in the neurotransmitter balance of this loop in PD with fatigue may result from changes in the receptor sensitivities of basal ganglia neurotransmitters (dopamine, acetylcholine, serotonin, adenosine) and defects in the transporter systems for dopamine and/or norepinephrine (16, 17). However, there is inconsistent evidence on the role of dopaminergic system in the pathogenesis of PD related fatigue (15, 17–20). Meanwhile, considering that fatigue is usually not alleviated after dopaminergic replacement (8), serotoninergic, glutamatergic (21) and other therapies (22), a few researchers have gradually focused their fatigue research on cholinergic aspects (23). Cholinergic degeneration is an important contributor to a number of clinical features of PD (24–27). More readily identification of PD patients with cholinergic system degeneration may possibly allow future targeted cholinergic treatment approaches, in addition to dopaminergic therapy, to ameliorate a diverse spectrum of nonmotor and motor clinical morbidity (28). Treatment with cholinesterase inhibitor drugs has shown improvement in fatigue in patients with PD with dementia (23). Whereas, another acetylcholinesterase [11C]-PMP PET study showed that neither thalamic nor cortical acetylcholinesterase binding was a significant predictor of fatigue in PD (17). Currently, the possible implication of cholinergic dysfunction in fatigue has not been thoroughly investigated.

The cholinergic basal forebrain (CBFB) is the most extensively studied and best characterized CNS cholinergic projection system (29). Basal forebrain degeneration produces cortical cholinergic denervation. Volumetric or density analysis of the CBFB on high-resolution structural MRI scans is available as an *in vivo* surrogate measure of cholinergic degeneration in normal aging and disease (30). To date, no imaging studies either in PD or in other neurological disorders, have explored the role of CBFB in the pathophysiology of fatigue.

Based on the cholinergic hypothesis, we proposed that central fatigue in PD may be associated with structural alterations in the CBFB. To test this, voxel-based morphometry (VBM) was applied to analyze structural MRI data, with gray matter density (GMD) and GMV serving as respective indicators of local tissue concentration and regional volume. Multiple linear regression analyses were further conducted to examine the relationships between fatigue severity and GMD/GMV in bilateral cholinergic nucleus 4 (Ch4) and 123 (Ch123). This methodological approach provides neuroimaging evidence regarding the cholinergic basis of central fatigue in PD.

## 2 Materials and methods

### 2.1 Participants

This retrospective investigation enrolled a cohort of 49 right-handed individuals diagnosed with idiopathic Parkinson’s disease (PD) in accordance with the Movement Disorder Society (MDS) clinical diagnostic criteria. Participants were recruited from Ningbo No.2 Hospital between March 2020 and July 2022. Eligibility for inclusion required: (1) PD diagnosis confirmation by a neurologist specializing in movement disorders, (2) completed Fatigue Severity Scale (FSS) evaluation, and (3) availability of structural brain MRI scans acquired during the same assessment period.

Exclusion criteria were systematically applied to minimize confounding effects, excluding individuals with: (1) major depressive disorder per Diagnostic and Statistical Manual of Mental Disorders (DSM-5) criteria; (2) PD-associated dementia based on established guidelines (31); (3) documented history of significant head trauma or cerebrovascular pathology; (4) hereditary, atypical, or secondary parkinsonism, or psychotic disorders; (5) excessive daytime somnolence (Epworth Sleepiness Scale score >10) (32); and (6) fatigue determined to be secondary to medication adverse effects.

### 2.2 Clinical data assessment

All patients’ demographic characteristics were collected. Motor severity and disease stage of each patient were evaluated with the MDS Unified Parkinson’s Disease Rating Scale (MDS-UPDRS) motor part III and Hoehn and Yahr (H-Y) stage, respectively. Neuropsychiatric assessment included the Mini-Mental State Examination (MMSE) to measure global cognitive function, the 24-item Hamilton Depression Rating Scale (HMAD-24) to assess depression, the Hamilton Anxiety Scale (HAMA) to assess anxiety, and the Epworth Sleeping Scale (ESS) to estimate daytime somnolence. Levodopa equivalent daily dose (LEDD) was calculated for all patients. Evaluations were conducted in the OFF-medication state to minimize pharmacologic confounding.

Fatigue was assessed using a two-step approach. Central fatigue was initially screened via Item 13 of MDS-UPDRS Part I, which inquires about frequent tiredness unrelated to sleepiness or low mood. Fatigue severity was then measured using the Fatigue Severity Scale (FSS) (33), a 9-item self-report instrument that evaluates physical, mental, and social dimensions of fatigue over the preceding two-week period (34). Items are rated on a 7-point Likert scale (1 = strongly disagree to 7 = strongly agree), with total scores ranging from 9 to 63; higher scores reflect greater fatigue severity. It should be noted that although the FSS is widely recommended for PD fatigue assessment (1), it may capture overlapping aspects of other non-motor symptoms.

### 2.3 Image acquisition

All MRI examinations were performed with a 3.0 T Siemens scanner (Trio, Erlangen, German) in the Ningbo No.2 Hospital. Foam pads were used to minimize head motion. The 3D high resolution T1 weighted structural images were obtained with magnetization prepared rapid gradient-echo sequence: echo time (TE)/repetition time (TR) = 2.10/1900 ms, field of view (FOV) = 240 mm × 240 mm, slice thickness = 1 mm, voxel size = 1 × 1 × 1 mm3, number of slices = 188.

### 2.4 VBM analysis

**Figure 1:**
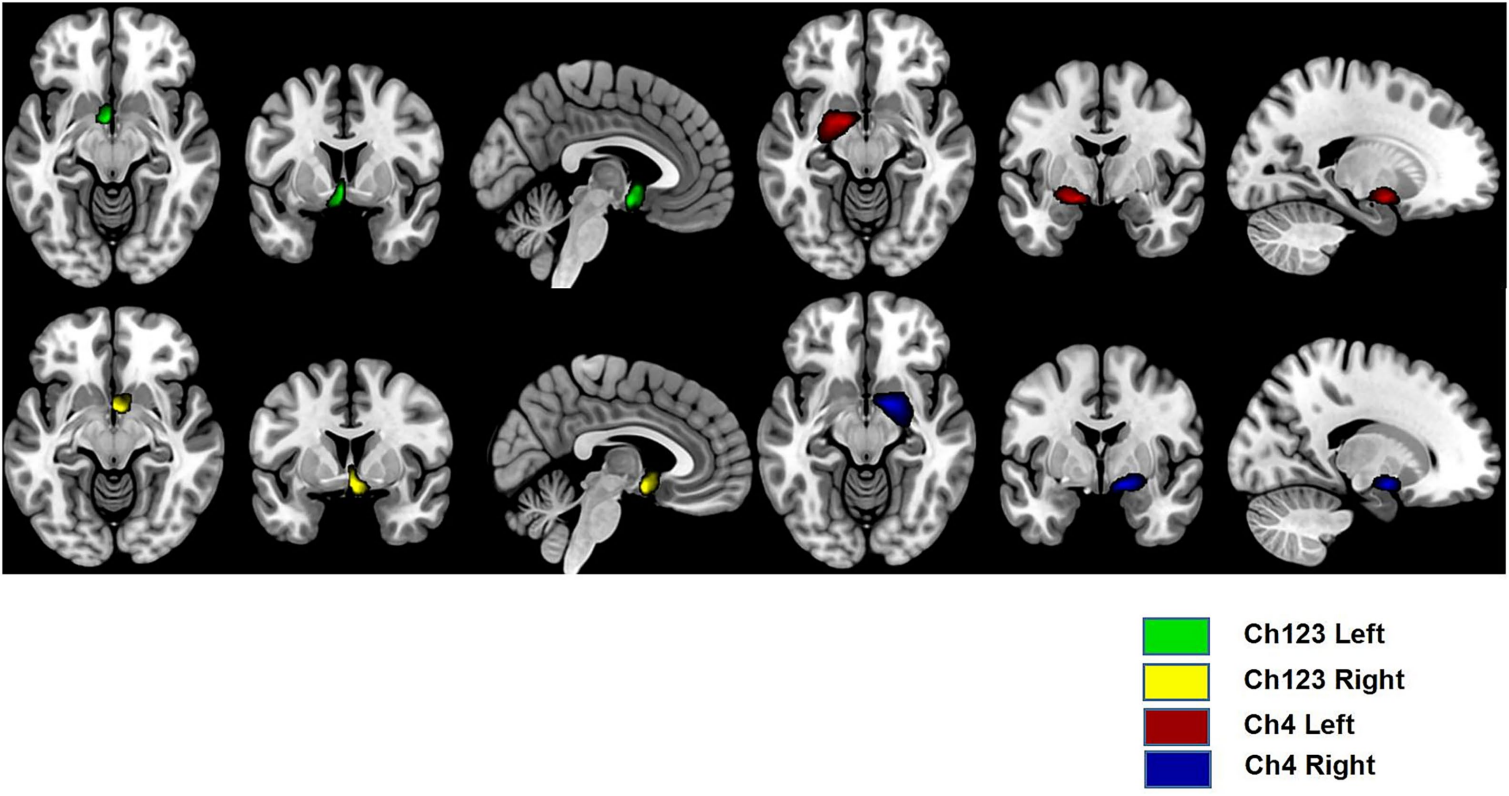
Visualization of the Ch123 and Ch4 in both hemispheres.

The neuroimaging procedures for quantifying the GMD and GMV of Ch4 and Ch123 regions have been previously reported in detail (35). GMD primarily reflects the local concentration of gray matter tissue at the voxel level, which can be influenced by microstructural features such as synaptic density and neuropil. In contrast, GMV represents the total volume of a gray matter structure, integrating information from both tissue density and its spatial extent. Although these measures are mathematically related, they provide complementary perspectives on gray matter integrity. Preprocessing of the structural images was conducted using FSL version 6.0.3. Subsequent analyses were implemented in a pipeline integrating MATLAB (MathWorks, Natick, MA, USA) with Statistical Parametric Mapping (SPM12) and the Computational Anatomy Toolbox (CAT12). This workflow enabled VBM of T1-weighted MPRAGE sequences. The CAT12 pipeline automatically performed tissue classification, segmenting each image into gray matter, white matter, and cerebrospinal fluid. Nonlinear spatial normalization to Montreal Neurological Institute (MNI) space was achieved via the Diffeomorphic Anatomical Registration Through Exponentiated Lie Algebra (DARTEL) approach (36). Modulation of gray matter volume maps was carried out by applying the Jacobian determinants derived from the normalization process. Region-of-interest (ROI) masks for Ch123 and Ch4, based on existing probability maps (Figure 1), were resampled to align with the resolution of the processed VBM data (24, 37). Using the Restplus toolkit, mean GMD values were extracted from each ROI (38). Total GMV for Ch123 and Ch4 was computed as the sum of all voxel values within the respective masks. Total intracranial volume (TIV) was automatically calculated within the CAT12 pipeline as the sum of gray matter, white matter, and cerebrospinal fluid volumes from the segmented, non-modulated images. To account for variations in head size, all regional GMV values for the basal forebrain were normalized using a proportional scaling approach, whereby each individual’s regional GMV was divided by their TIV and then multiplied by the sample mean TIV. This yielded normalized GMV values suitable for group-level comparisons (39).

### 2.5 Statistical analyses

Shapiro normality test was used to determine the normality of the sample data for continuous (quantitative) data. Mean ± standard deviation was used to represent the sample data if it met the normal distribution; median (interquartile) was used to represent the sample data if it did not meet the normal distribution; and frequency (percentage) was used to describe the classified (qualitative) data statistically. Spearman correlation analyses were used to evaluate the crude associations of FSS and GMD/GMV. To ensure a statistically robust test of our primary hypothesis, the analysis focused on a parsimonious model. Although extensive clinical data were collected for characterization, including all measures as covariates was precluded by our sample size to avoid overfitting and preserve statistical power for the key variables of interest. In the PD cohort, voxel-based multiple linear regression analyses (based on general linear model) were performed to map FSS effects on GMD and GMV. FSS values served as dependent variables. Shapiro–wilk test was used to check the distribution of the residuals of multiple linear regression models. For those residuals not normally distributed, data log2 or box-cox data transformation were performed. In addition, age and sex were used as external regressions to control their effects on both brain GMD and FSS, and further investigated the presence and specificity of the association between GMV and FSS when adjusting for age, sex, and TIV. For all analyses, *a priori p-*values of <0.05 were considered statistically significant.

## 3 Results

### 3.1 Participants characteristics and scale values

The characteristics of all patients are presented in Table 1. Subjects ranged in age from 47 to 80 years and included 26 men (53.1%) and 23 women (46.9%). Patients with PD exhibited mild to severe motor symptoms (MDS-UPDRS III total score: 37, range: 14–85) and early to late disease stages (H-Y mean stage: 2.0, range: 1–4). The median LEDD was 309.38 mg (0–1275.6). The median score of MMSE was 26 (25–30), HAMD was 8.5 (0–32), HAMA was 8 (0–28) and ESS was 3 (0–20). The median score of total FSS was 9 (9–63).

**Table 1:** Demographic and clinical characteristics of PD patients.

Variables	*N*	Value
Number of subjects, *n*	49	49
Women/Men, *n* (%)/*n* (%)	49	23 (46.9%)/26 (53.1%)
Age at MRI, mean years (±SD)	49	64.6 ± 8.6
Years of education, median (IQR)	48	6.0 (6.3)
Duration of disease, median months (IQR)	48	49.0 (67.5)
MDS-UPDRS, Part 1, total score, median (IQR)	49	8.0 (9.0)
MDS-UPDRS, Part 2, total score, median (±SD)	49	14.3 ± 7.4
MDS-UPDRS, Part 3, off score, median (IQR)	49	37.0 (19.0)
Hoehn and Yahr, median (IQR)	49	2 (1)
LEDD, median (IQR)	44	309.4 (587.5)
HAMD score, median (IQR)	48	8.5 (13.0)
HAMA score, median (IQR)	48	8.0 (10.8)
MMSE score, median (IQR)	48	26.0 (5.3)
FSS total score, median (IQR)	49	9 (27)
ESS score, median (IQR)	49	3.0 (7.0)

SD, standard deviation; IQR, interquartile range; LEDD, Levodopa equivalent daily dose; HAMD, 24-item Hamilton Depression Rating Scale; ESS, Epworth Sleepiness Scale; FSS, Fatigue Severity Scale; MDS-UPDRS, Movement Disorder Society Unified Parkinson’s Disease Rating Scale.

### 3.2 Association between structural signatures of Ch4/Ch123 and FSS

We performed a comprehensive analysis of structural changes in the bilateral Ch4 and Ch123 regions in patients with PD. The GMD and GMV values for all ROIs - including bilateral total, left, and right Ch4 and Ch123 - are summarized in Table 2.

**Table 2:** Grey matter density and grey matter volume values of Ch4/Ch123.

Variables	*N*	Value
Regional standardized gray matter density, mean (±SD)		
Ch4_total	49	0.4051 ± 0.0683
Ch4_left	49	0.3803 ± 0.0401
Ch4_right	49	0.4215 ± 0.0532
Ch123_total	49	0.5324 ± 0.0768
Ch123_left	49	0.6304 ± 0.0910
Ch123_right	49	0.4625 ± 0.0773
Regional standardized gray matter volume, mean (±SD)		
Ch4_total	49	71.7020 ± 12.0902
Ch4_left	49	32.3217 ± 3.4060
Ch4_right	49	38.7758 ± 4.8916
Ch123_total	49	85.7130 ± 12.3708
Ch123_left	49	42.2369 ± 6.0937
Ch123_right	49	43.4761 ± 7.2657
TIV	49	1521.3363 ± 160.7294

Ch4, Cholinergic nucleus 4; Ch123, Cholinergic nuclei 1, 2 and 3; TIV, total intracranial volume.

**Figure 2:**
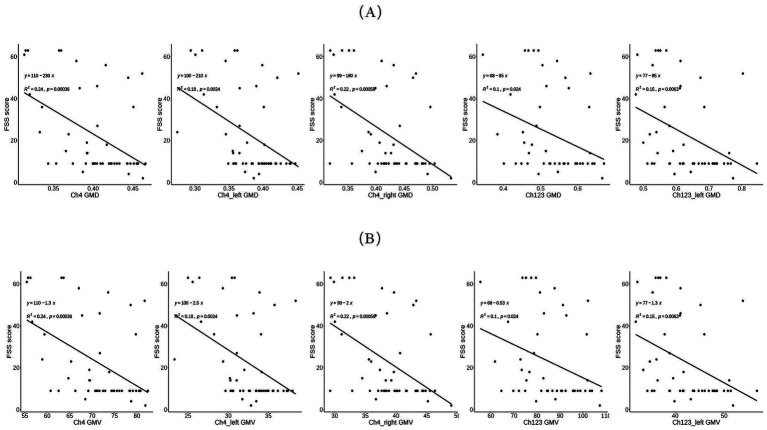
Scatter plot of correlation between Fatigue Severity Scale and **(A)** Density of different regions of interest Parkinson’s disease patients, and **(B)** Volume of them.

We first employed Spearman correlation analysis to preliminarily examine the associations between fatigue severity (FSS) and GMD/GMV across these regions. As illustrated in Figure 2, most regions showed negative correlations with FSS, with the exception of the right Ch123.

Subsequent multiple linear regression analyses, adjusted for age and sex, revealed that higher FSS scores were significantly associated with reduced GMD in the total, left, and right Ch4 regions. A similar pattern was observed for the total and left Ch123 GMD (Table 3).

**Table 3:** Association between FSS score and GMD values of Ch4/Ch123 in PD patients.

FSS	Regions	*B*	SE	*t*	*p*	Lower 95%CI	Upper 95%CI
Ch4	Total	−8.259	2.933	−2.815	0.007	−14.010	−2.510
	Left	−6.789	3.094	−2.194	0.033	−12.850	−0.720
	Right	−6.486	2.387	−2.718	0.009	−11.160	−1.810
Ch123	Total	−4.397	1.876	−2.344	0.024	−8.070	−0.720
	Left	−3.729	1.559	−2.392	0.021	−6.790	−0.670
	Right	−3.232	1.794	−1.802	0.078	−6.750	0.280

Multiple linear regression was used to examine the age- and sex-adjusted associations between FSS score and the values of GMD.

Ch4, Cholinergic nucleus 4; Ch123, Cholinergic nuclei 1, 2 and 3; PD, Parkinson’s disease; GMD, gray matter density.

When further controlling for total intracranial volume (TIV) in addition to age and sex, higher fatigue levels remained significantly associated with smaller GMV in both the total and right Ch4 regions (Table 4). In contrast, no significant association was found between Ch123 GMV and FSS scores.

**Table 4:** Association between FSS score and GMV values of Ch4/Ch123 in PD patients.

FSS	Regions	*B*	SE	*t*	*p*	Lower 95%CI	Upper 95%CI
Ch4	Total	−0.044	0.017	−2.575	0.013	−0.080	−0.010
	Left	−0.073	0.037	−1.954	0.057	−0.150	−0.000
	Right	−0.066	0.026	−2.516	0.016	−0.120	−0.010
Ch123	Total	−0.025	0.013	−1.953	0.057	−0.050	0.000
	Left	−0.051	0.026	−1.993	0.052	−0.100	0.000
	Right	−0.029	0.020	−1.468	0.149	−0.070	0.010

Multiple linear regression was used to examine the age-, sex- and TIV-adjusted associations between FSS score and the values of GMV.

Ch4, Cholinergic nucleus 4; Ch123, Cholinergic nuclei 1, 2 and 3; PD, Parkinson’s disease; GMV, gray matter volume; TIV, total intracranial volume.

## 4 Discussion

In this study, we investigated the plausible yet under-explored relationship between degeneration of the CBFB - the primary source of cerebral cholinergic input - and central fatigue in PD, using a prior region-of-interest approach based on VBM. Results revealed a significant negative association between fatigue severity and both GMD and GMV in the CBFB. Specifically, central fatigue was negatively correlated with GMD and GMV in bilateral Ch4 subregions, as well as with GMD in the left Ch123 complex. These findings align with the previously proposed hypothesis that structural alterations in the CBFB contribute to central fatigue in PD. By extending earlier neuroanatomical studies on fatigue-related brain changes, our results provide new insights into the brain-behavior relationship underlying central fatigue and its potential cholinergic mechanisms in PD.

The cholinergic pathways originating from the basal forebrain exhibit distinct connectivity patterns (40), particularly through the Ch4 neuronal group within the nucleus basalis of Meynert, which projects broadly across the cerebral cortex (41) and participates in attention, memory, and effort-related behavioral processes (28, 42, 43). These anatomical and functional characteristics offer a plausible substrate for understanding the relationship between central fatigue and cholinergic basal forebrain integrity. Clinically, cholinergic deficiency in Parkinson’s disease has been associated not only with cognitive decline (24, 37) but also with postural and gait impairment (39, 44), sleep disturbances (37), apathy (42), and autonomic dysfunction (27)—features often resistant to dopaminergic treatment and indicative of a malignant hypocholinergic PD subtype (28, 45). Notably, central fatigue manifests as diminished capacity to sustain mental effort and attention (46–49), overlapping functionally with domains modulated by cholinergic circuits. Supporting this, neuroimaging studies have identified altered activity and connectivity within attention-related networks, such as reduced low-frequency fluctuations in the right middle frontal gyrus and disrupted fronto-parieto-temporal functional connectivity, in fatigued PD patients (50). Given the considerable symptomatic and mechanistic overlap between central fatigue and the central cholinergic deficiency syndrome, it is reasonable to hypothesize that fatigue may represent a clinical manifestation within this broader cholinergic spectrum.

In this study, we employed a region-specific analytical approach to evaluate the relationship between CBFB integrity and central fatigue, examining both GMD and GMV as distinct yet complementary indices. Given the anticipated high collinearity between GMD and GMV in small subcortical nuclei such as Ch4 and Ch123, confirmed by post-hoc correlation analysis, we performed parallel regression analyses for both metrics rather than selecting a single indicator. This methodological strategy allowed a comprehensive assessment of structural-fatigue associations.

The results revealed consistent negative associations between fatigue severity and both GMD and GMV in bilateral Ch4, supporting the primary conclusion that structural degeneration of this cortical-projecting cholinergic nucleus underlies fatigue in PD (29). A weaker but significant correlation was also observed between fatigue and left Ch123 GMD, suggesting a partial role of this hippocampal- and olfactory-projecting complex (51), potentially mediated by cognitive mechanism (29). However, several other cholinergic studies did not find an association between Ch123 GMD and specific symptoms (39, 42, 44). No association was found with Ch123 GMV. Importantly, the convergent findings from both GMD and GMV analyses strengthen the robustness of the central Ch4-fatigue association, which emerged as more prominent than Ch123 effects, with GMD offering slightly more definitive evidence than GMV.

Current evidence regarding CBFB volume changes in neurodegenerative diseases remains inconsistent. For instance, transient volume increases in this region have been observed during the progression of Alzheimer’s disease (52). In PD associated with leucine-rich repeat kinase 2 (LRRK2) mutations, preliminary acetylcholinesterase PET data indicate elevated or hypercholinergic activity (53). Such alterations may reflect neuroplastic compensation, amyloid pathology, neuroinflammatory processes, or other mechanisms (52). Further supporting this pattern, LRRK2 mutations have been linked to enlarged CBFB volume (54). Given that LRRK2-related PD typically presents with a milder phenotype, less cognitive decline, and improved prognosis, CBFB expansion may represent an adaptive cholinergic response (54). Interestingly, LRRK2 variant carriers also demonstrate higher susceptibility to fatigue compared to non-carriers (55). Collectively, these observations align more closely with the recently proposed “compensatory” hypothesis of dopaminergic–cholinergic interplay than with purely neuroinflammatory explanations. According to the compensatory framework, dysregulation of both dopaminergic and cholinergic systems can intensify central fatigue. In the early stage of PD, concurrent dysfunction in both neurotransmitter systems triggers cholinergic upregulation and volumetric expansion of the CBFB. However, as cholinergic function progressively declines, this compensatory mechanism fails, leading to worsening fatigue, slowed gait, cognitive deficits, and other features of a malignant PD subtype. Experimental studies comparing dual-system and single-system lesions have provided supporting evidence for this dopaminergic–cholinergic interaction model (56).

This study offers neuroimaging support for the cholinergic hypothesis of central fatigue in PD; however, several limitations should be acknowledged. First, while our findings establish a significant association between CBFB integrity and fatigue, the complex pathophysiology of fatigue likely involves multiple neural circuits and neurotransmitter systems beyond the cholinergic system. Our cross-sectional design and model parsimony, while focused on testing a specific cholinergic hypothesis, preclude definitive conclusions regarding potential mediation effects through other factors such as motor severity or specific cognitive deficits. Future studies with larger sample sizes and comprehensive multimodal assessments are needed to disentangle the independent contribution of cholinergic degeneration from other interrelated pathological processes in PD-related fatigue. The absence of a healthy control group limits the pathological specificity of the observed associations between gray matter integrity and fatigue. Future case–control designs are needed to confirm whether these relationships are unique to PD. Additionally, the modest sample size may constrain statistical power, and the restriction of anatomical analysis to partial CBFB regions offers only an indirect reflection of cholinergic integrity due to the cellular heterogeneity of this area (57, 58). Methodologically, the use of MMSE rather than Montreal Cognitive Assessment (MoCA) may have reduced sensitivity in detecting PD-specific cognitive deficits. Furthermore, the complex pathophysiology of fatigue likely involves multiple neural circuits and neurotransmitter systems beyond the cholinergic system. Future studies with larger cohorts should examine cholinergic volume changes in relation to disease duration, clinical phenotypes, and genotype variations. It would also be valuable to explore the independent contribution of cholinergic deficits to fatigue and assess the potential of cholinergic augmentation therapies. In this context, Ch4 GMD/GMV could serve as a potential biomarker for selecting patients who may benefit from such interventions.

## 5 Conclusion

In conclusion, our findings represent the first known neuroimaging evidence of an association between CBFB degeneration and central fatigue in patients with PD. Central fatigue was negatively correlated with bilateral Ch4 GMD/GMV and with left-sided Ch123 GMD.

Our findings suggest that the structural degradation of CBFB is involved in the central fatigue of PD, providing new ideas and neuroimaging evidence for the cholinergic theory of central fatigue in PD.

## Data Availability

The raw data supporting the conclusions of this article will be made available by the authors, without undue reservation.

## Glossary

CBFB: Cholinergic basal forebrain
PD: Parkinson’s disease
MRI: Magnetic resonance imaging
FSS: Fatigue Severity Scale
GMD: Gray matter density
GMV: Gray matter volume
Ch4/123: Cholinergic nucleus 4/123
TIV: Total intracranial volume
MPRAGE: Magnetization prepared rapid acquisition gradient echo
VBM: Voxel-based morphometry
MDS: Movement Disorder Society
MDS-UPDRS: Movement Disorder Society Unified Parkinson’s Disease Rating Scale
fMRI: Functional magnetic resonance imaging
H-Y: Hoehn and Yahr
MMSE: Mini-Mental State Examination
HAMD-24: Hamilton Depression Rating Scale-24
HAMA: Hamilton Anxiety Rating Scale
ESS: Epworth Sleepiness Scale
LEDD: Levodopa equivalent daily dose
MoCA: Montreal Cognitive Assessment
TE: Echo time
TR: Repetition time
FOV: Field of view
SPM: Statistical Parametric Mapping
CAT: Computational Anatomy Toolbox
MNI: Montreal Neurological Institute
ROI: Regions of interest
LRRK2: Leucine-rich repeat kinase 2
